# Plastidial Starch Phosphorylase in Sweet Potato Roots Is Proteolytically Modified by Protein-Protein Interaction with the 20S Proteasome

**DOI:** 10.1371/journal.pone.0035336

**Published:** 2012-04-10

**Authors:** Yi-Chen Lin, Han-Min Chen, I-Min Chou, An-Na Chen, Chia-Pei Chen, Guang-Huar Young, Chi-Tsai Lin, Chiung-Hsiang Cheng, Shih-Chung Chang, Rong-Huay Juang

**Affiliations:** 1 Department of Biochemical Science and Technology, and Institute of Microbiology and Biochemistry, National Taiwan University, Taipei, Taiwan; 2 Department of Life Science, and Institute of Applied Science and Engineering, Catholic Fu-Jen University, Taipei, Taiwan; 3 Division of Nephrology, Department of Internal Medicine, National Taiwan University Hospital, Taipei, Taiwan; 4 Institute of Bioscience and Biotechnology, and Marine Center for Bioscience and Biotechnology, National Taiwan Ocean University, Keelung, Taiwan; 5 Animal Cancer Research Center, Department of Veterinary Medicine, School of Veterinary Medicine, National Taiwan University, Taipei, Taiwan; Karolinska Institutet, Sweden

## Abstract

Post-translational regulation plays an important role in cellular metabolism. Earlier studies showed that the activity of plastidial starch phosphorylase (Pho1) may be regulated by proteolytic modification. During the purification of Pho1 from sweet potato roots, we observed an unknown high molecular weight complex (HX) showing Pho1 activity. The two-dimensional gel electrophoresis, mass spectrometry, and reverse immunoprecipitation analyses showed that HX is composed of Pho1 and the 20S proteasome. Incubating sweet potato roots at 45°C triggers a stepwise degradation of Pho1; however, the degradation process can be partially inhibited by specific proteasome inhibitor MG132. The proteolytically modified Pho1 displays a lower binding affinity toward glucose 1-phosphate and a reduced starch-synthesizing activity. This study suggests that the 20S proteasome interacts with Pho1 and is involved in the regulation of the catalytic activity of Pho1 in sweet potato roots under heat stress conditions.

## Introduction

Starch is the main storage polysaccharide in plants. Several essential enzymes are involved in starch biosynthesis, including ADP-glucose pyrophosphorylase, starch synthase, branching enzyme, and debranching enzyme [1]. In higher plants, starch phosphorylase (Pho, or SP, EC 2.4.1.1) plays a key role in starch metabolism [2]–[5]. Pho catalyzes the reversible phosphorolysis of starch and produces glucose 1-phosphate (Glc-1-P) as one of its products [6], [7]. However, the biochemical mechanism that regulates whether Pho mediates degradation or synthesis of starch is unclear. Plants express different types of Pho, which are classified as low-affinity type (Pho1, L-form SP, or L-SP) and high-affinity type (Pho2, H-form SP, or H-SP), according to their binding affinities toward starch [8]–[10]. Notably, an insert sequence containing 78 amino acids (L78) was found uniquely in the middle of the Pho1 molecule, though not in Pho2. This insertion, located near the glucan binding site, is believed to cause a steric hindrance and prevents Pho1 from binding to polyglucan substrates effectively [11].

Previous studies showed that the accumulation of starch is proportional to the expression and activity of Pho1 in potato tubers [12]–[16], maize endosperm [17], rice [18], [19], wheat [4], sweet potato roots [20], spinach [14], and pea [21]. Such data suggest that Pho1 is associated with starch biosynthesis. Moreover, Satoh et al. [3] found that a mutation in the *Pho1* gene of *oryza sativa* significantly affected the starch content and the size of mature seeds at 20°C, indicating that Pho1 may be required for normal starch biosynthesis in rice endosperm at low temperatures. However, an *Arabidopsis thaliana* mutant deficient in the *Atphs1* gene, a homolog of *Pho1*, showed no evident change in starch biosynthesis or degradation, but was more sensitive to abiotic stress. These observations suggest that *Atphs1* may be required for stress tolerance, and imply that Pho1 plays a vital role under certain environmental conditions [22].

Due to the significance of Pho1 in higher plants, some attention has been given to the regulation of its activity. Most of the Pho1 isolated from mature potato tubers [13] or sweet potato roots [23] was proteolytically modified and showed an intact 110 kDa band (P110) and a group of proteolytic bands (F50s) that are approximately 50 kDa on the SDS-PAGE. Thus, L78 in the central region of Pho1 has been proposed to be the proteolytic site. Interestingly, the proteolytically modified Pho1 still retains its quaternary structure and remains functionally active. Our previous studies using amino acid sequencing and the specific monoclonal antibody against the N- or C-terminal fragment of Pho1 identified three major cutting sites on L78 [24]. Additionally, the L78 sequences also contain many unique features, including potential phosphorylation sites, a polyproline II helix, and PEST regions which are rich in proline (P), glutamic acid (E), serine (S), and threonine (T) [24]. Tetlow et al. [25] reported that wheat Pho1 was phosphorylated and could form multiprotein complexes with the phosphorylated starch branching enzymes (SBEI and SBEIIb). Our group also found that Pho1 from sweet potato roots was phosphorylated *in vitro*, and that the phosphorylated Pho1 showed higher sensitivity to proteolytic modification [26]. These findings suggest that some regulatory mechanisms may control Pho1’s activity through protein-protein interaction or proteolytic modification. However, it remains unclear what mechanism modulates Pho1’s degradation.

The ubiquitin-proteasome system is a tightly regulated pathway that controls protein levels in eukaryotes [27]–[29]. Glucose-6-phosphate dehydrogenase [30], glutamine synthetase [30]–[32], fructose-1,6-bisphosphatase [31], malate dehydrogenase [33], and sucrose synthase [34], [35] have been shown to be degraded by the proteasome-mediated degradation pathway. The 26S proteasome consists of a 20S catalytic core and one or two 19S regulatory particles capping the core at either or both ends. Proteins targeted for degradation are conjugated with ubiquitin by an E1-E2-E3 enzymatic cascade. The ubiquitinated proteins are then recognized and degraded by the 26S proteasome in an ATP-dependent manner [29]. In plants, the 26S proteasome has been implicated in cell cycle progression, hormone responses, photomorphogenesis, plant development, and pathogen resistance by the selective removal of short-lived regulatory proteins [29]. The 20S proteasome has a cylindrical structure, which is highly conserved throughout prokaryotes and eukaryotes. Its catalytic sites are located in the inner chamber of the 20S particle and are only accessible via narrow entrances. Accumulated data have demonstrated that the 20S proteasome can cleave proteins in an ATP- and ubiquitin-independent manner [36], [37].

In this study we present evidence that the 20S proteasome associates with Pho1 and is specifically involved in the degradation of the L78 insertion in sweet potato roots. Our data also demonstrated that incubating sweet potato roots at 45°C accelerated the MG132 inhibited degradation of the L78 insertion by the 20S proteasome. Furthermore, the kinetic properties of Pho1 were altered after heat treatment, resulting in the proteolytic modification of Pho1. This study reveals that the 20S proteasome is involved in the regulation of Pho1 catalytic activity.

## Results

### A Complex Formed by the Interaction between Pho1 and the 20S Proteasome was Isolated from Sweet Potato Roots

**Figure 1:**
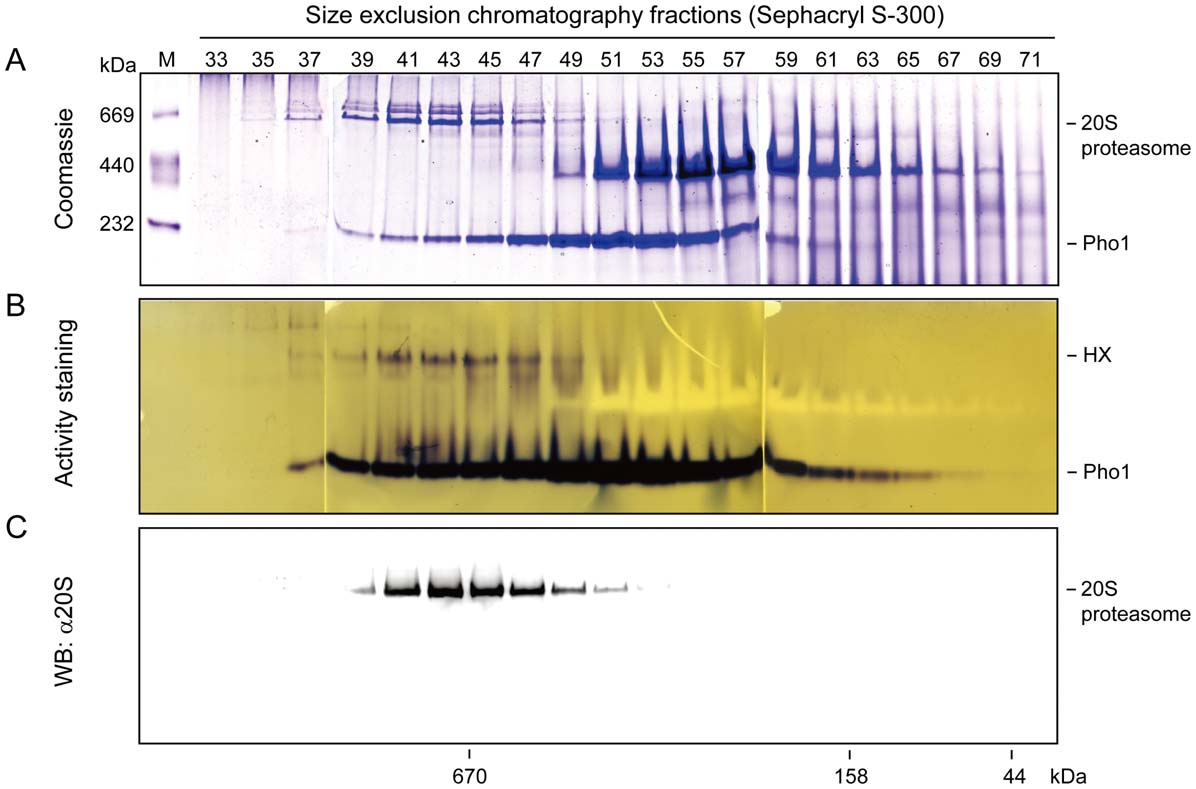
A high molecular weight complex (HX) showing Pho1 activity was found in the sweet potato roots. (**A**) The protein fraction from the extract of sweet potato roots was resolved by size-exclusion chromatography (Sephacryl S-300). The fractions (nos. 33∼71) after chromatography were analyzed by 6% native PAGE, and then stained with Coomassie Brilliant Blue R-250 (Coomassie). The molecular mass markers (thyroglobulin (porcine thyroid ), 669 kDa; ferritin (equine spleen ), 440 kDa; catalase (bovine liver ), 232 kDa) were revealed in lane 1. (**B**) The native PAGE gel was incubated with the reaction buffer containing 20 mM MES-KOH (pH 5.9), 1.2% soluble starch and 32 mM Glc-1-P at 37°C for 2 h. The zymogram was detected using iodine staining to reveal the catalytic activity of Pho1 in the direction of starch synthesis. Both of Pho1 and HX showed starch-synthesizing activity. The bands with negative staining were identified as beta amylase (BA). (**C**) The native PAGE gel was transferred to PVDF membranes and subsequently analyzed by western boltting with anti-20S proteasome antibody (α20S). The molecular mass in kilodalton (kDa) was shown as indicated on the bottom of the figure by applying the calibration of the size-exclusion chromatography column with known molecular mass standards (thyroglobulin (bovine), 670 kDa; γ-globulin (bovine), 158 kDa; ovalbumin (chicken), 44 kDa).

**Figure 2:**
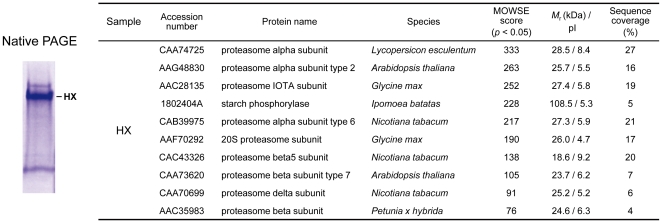
HX is composed of Pho1 and the 20S proteasome. The purified HX was separated by native PAGE and subsequently stained with Coomassie. The single HX band on native PAGE was sliced out for mass spectrometry analysis. The mass spectroscopic results (shown on the right) revealed that HX is composed of starch phosphorylase (Pho1) and several 20S proteasome subunits.

During the purification of Pho1 from sweet potato roots by size-exclusion chromatography (Fig. 1A), we found an unknown high molecular weight complex (HX) on native PAGE showing Pho1 activity after in-gel activity staining (Fig. 1B). To isolate this complex, fractions (nos. 37∼47 in Figure 1) containing HX were purified, analyzed by native PAGE, and subsequently examined by mass spectrometry analysis (Fig. 2). Surprisingly, the results showed that HX was composed of Pho1 and the 20S proteasome (Fig. 2). Therefore, we performed a western blotting with the anti-sweet potato root 20S proteasome antibody to detect the 20S proteasome in the fractions of the size-exclusion chromatography. The results showed that the 20S proteasome and HX appeared to co-purify in the overlapping fractions (Fig. 1C).

**Figure 3:**
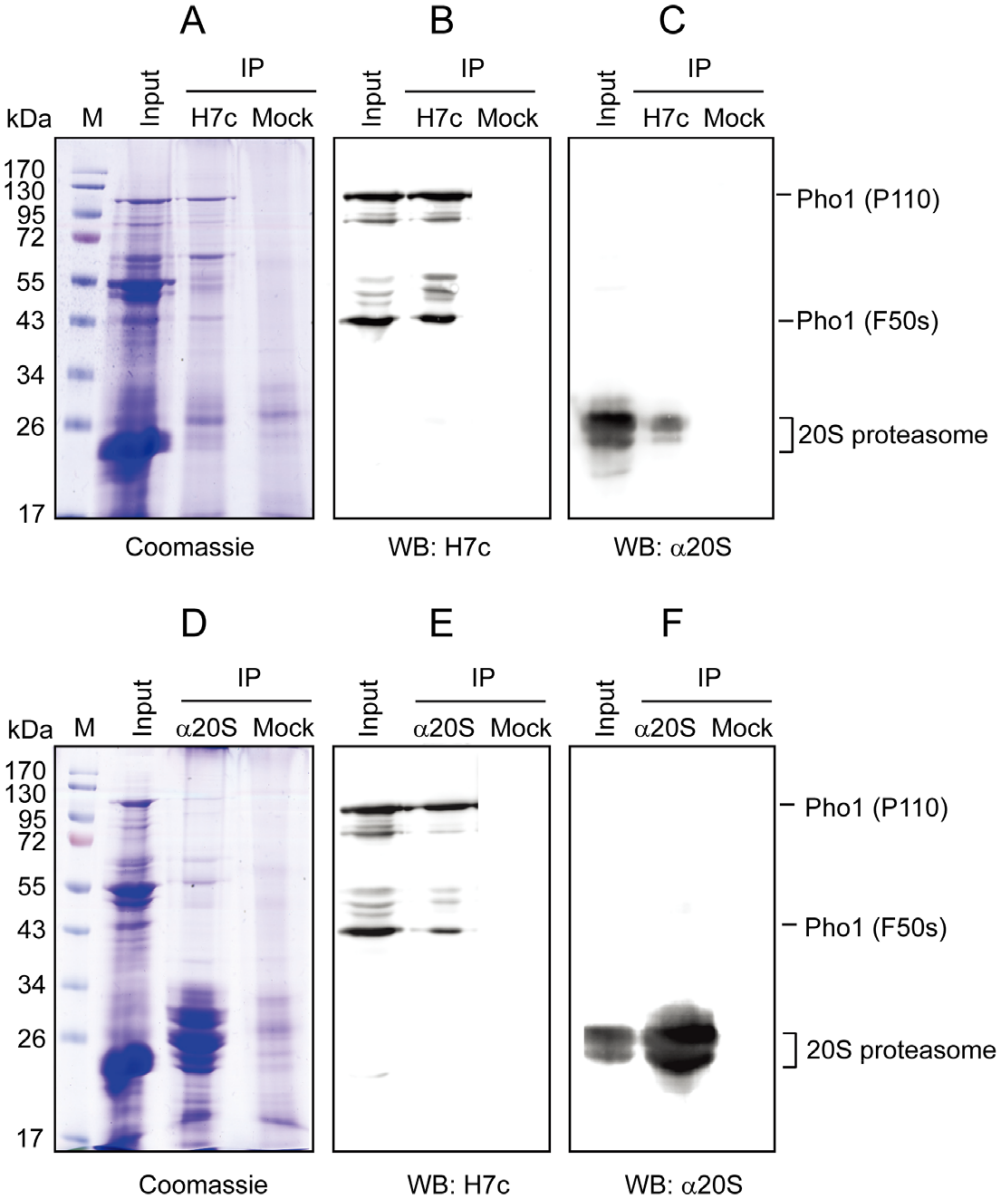
The 20S proteasome was co-immunoprecipitated by Pho1 specific monoclonal antibody H7c from whole cell extracts of sweet potato roots. Pho1 specific monoclonal antibody H7c-crosslinked Protein A Sepharose CL-4B beads were used to pull down Pho1 from the whole cell extracts in order to study the interaction between Pho1 and the 20S proteasome. After the immunoprecipitation procedures, beads were incubated with SDS-PAGE sample buffer at 100°C for 10 min and separated by 12.5% SDS-PAGE. The gels were either stained with Coomassie (**A**) or transferred to PVDF membranes for staining with H7c (**B**) and anti-20S proteasome (**C**) primary antibodies. One tenth of the input crude extracts (10 µg) was applied on the gel for comparison (Input). The control experiment was performed by replacing H7c with unrelated monoclonal antibody (Mock). Alternatively, anti-20S proteasome antibody-crosslinked Protein A Sepharose CL-4B beads were also used to pull down 20S proteasomes from whole cell extracts. After the immunoprecipitation procedures, the gels were either stained with Coomassie (**D**) or transferred to PVDF membranes for staining with H7c (**E**) and anti-20S proteasome (**F**) primary antibodies. M, prestained SDS-PAGE molecular weight standards.

To further confirm the interaction between Pho1 and the 20S proteasome, a Pho1 specific monoclonal antibody, H7c, was used to immunoprecipitate Pho1 from the crude extract of sweet potato roots. The immunoprecipitant was separated by SDS-PAGE (Fig. 3A), and analyzed by western blotting using H7c (Fig. 3B) and anti-20S proteasome primary antibodies (Fig. 3C), respectively. The data showed that a group of protein bands around 27∼31 kDa, which were co-immunoprecipitated by H7c, were clearly detected by anti-20S proteasome antibody (Fig. 3C, lane 2). Alternatively, we also used anti-20S proteasome antibodies (α20S) to co-immunoprecipitate possible the 20S proteasome-interacting proteins from the same crude extract sample. The data showed that Pho1 (containing P110 and F50s) was clearly detected by H7c in the sample co-immunoprecipitated by α20S (Fig. 3E, lane 2). The result suggests that Pho1 is likely associated with the 20S proteasome.

### The N-terminal Domain and the L78 Insertion of Pho1 Interact with the 20S Proteasome

**Figure 4:**
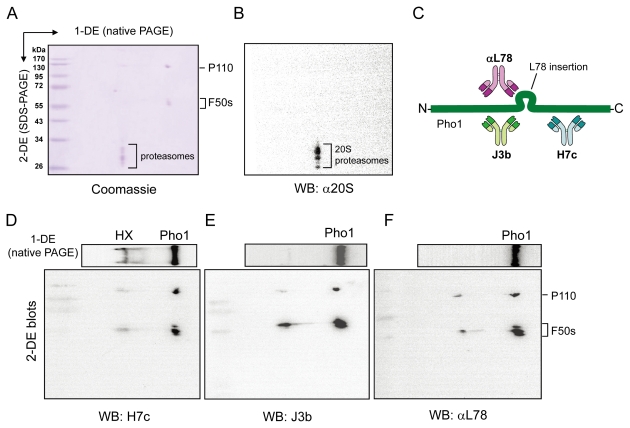
The 20S proteasome interacts with the N-terminal domain and L78 insertion of Pho1. To obtain a better electrophoresis resolution, HX was separated by native PAGE for the first dimension (1-DE) and then subsequently applied to the second-dimension SDS-PAGE (2-DE). (**A**) The 2-DE gel was stained with Coomassie to detect P110, F50s and multiple 20S proteasome subunits. (**B**) The 2-DE gel was transferred to a PVDF membrane and analyzed by western blotting using the rabbit anti-sweet potato root 20S proteasome antiserum. (**C**) The simple diagram showed the corresponding recognition site of J3b, H7c or αL78 antibody on Pho1. The letters “N” and “C” indicate the N terminus and the C terminus of Pho1, respectively. To investigate the interaction regions between Pho1 and the 20S proteasome, the 1-DE gels and the 2-DE gels were transferred to PVDF membranes and analyzed by western blotting using H7c (**D**), J3b (**E**) and αL78 (**F**) primary antibodies. J3b and αL78 did not bind to the native form of HX.

To examine the components of HX in native and denatured conditions, a modified two-dimensional gel electrophoresis was applied to the experiment. HX was separated by native PAGE for the first dimension (1-DE) and then subsequently applied to the second-dimension SDS-PAGE (2-DE). The 2-DE gel stained with Coomassie Brilliant Blue R-250 (Coomassie) reveals that HX contains P110, F50s (Fig. 4A) and multiple 20S proteasome subunits that were also specifically recognized by the rabbit anti-sweet potato root 20S proteasome antiserum (Fig. 4A, 4B). In our previous studies, we prepared several antisera and monoclonal antibodies which could specifically recognize defined epitopes on the Pho1 molecule. Figure 4C shows the sites on Pho1 which are specifically recognized by J3b, H7c and αL78 monoclonal antibodies, respectively. To investigate the interaction regions between Pho1 and the 20S proteasome, the 1-DE gel and the 2-DE gel were transferred to PVDF membranes and analyzed by western blotting using H7c, J3b and αL78 primary antibodies (Fig. 4D, 4E, 4F). Intact P110 and the F50s fragments of Pho1 were clearly detected by these antibodies in the 2-DE gels (Fig. 4D, 4E, 4F, 2-DE blots). The H7c antibody detected the HX complex in the 1-DE blot (Fig. 4D, 1-DE, H7c). In contrast, J3b and αL78 did not detect the HX complex in the native condition (Fig. 4E, 4F, 1-DE, J3b and αL78), suggesting that J3b and αL78 antibody binding were hindered in the presence of the 20S proteasome. As the recognition epitopes for J3b and αL78 are contained in the N-terminal domain and the L78 insertion respectively, these observations imply that the 20S proteasome interacts with these regions of Pho1.

### Heat Treatment Promoted Pho1 Degradation at the L78 Insertion by the Proteasome

**Figure 5:**
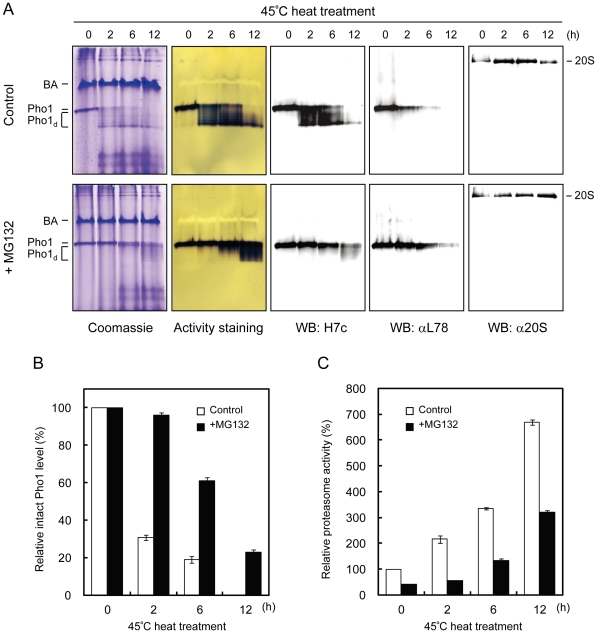
Heat treatment triggered the proteolytic modification of Pho1 by the 20S proteasome. In two parallel experiments sweet potato root discs (0.5 mm thick) were pretreated without (Control) or with 100 µM of MG132 (+MG132), and then incubated at 45°C for the indicated time periods. BA, beta amylase; Pho1, intact Pho1; Pho1_d_, proteolytic modified Pho1. (**A**) The crude protein extracts of the discs (10 µg) were separated by 7.5% native PAGE and analyzed by Coomassie staining, Pho1 activity staining or western blotting with H7c, αL78 or α20S antibody, respectively. Data are representative of three independent experiments. (**B**) Quantitative densitometry of the protein levels of intact Pho1 on the western blots detected by αL78 antibody in Figure 5A. The intact Pho1 level of the sample pretreated without (Control) or with MG132 (+MG132) at time zero was expressed as 100%. Values are means ± S.D. from three independent experiments. (**C**) The 20S proteasome activities in the samples pretreated without (Control, bars in white) or with MG132 (+MG132, bars in black) were also determined by using Suc-LLVY-AMC as the substrate. The 20S proteasome activity in the control sample at time zero was expressed as 100%. Values are means ± S.D. from three independent experiments.

The catalytic activity of Pho1 in sweet potato roots are modified by proteolysis [24], which is enhanced by phosphorylation at Ser-527 on the L78 insertion [26]. However, the mechanism by which the degradation of Pho1 is regulated and the factor triggering this degradation process remain unknown. In an attempt to answer these questions, we tested several environmental stress conditions, including heat treatment. We found that incubating sweet potato roots at 45°C caused stepwise degradation of Pho1 in a time-dependent manner (Fig. 5A). Partially degraded Pho1 (Pho1_d_) revealed as multiple bands on the native PAGE still displays the catalytic activity in the direction of starch synthesis within 6 h of incubation (Fig. 5A, Control, Activity staining). After 12 h of incubation at 45°C, the enzyme activity of Pho1 decreased significantly, and the intact Pho1 was not detected by H7c antibody on the western blot (Fig. 5A, Control, WB: H7c). Moreover, the L78 insertion was also completely removed as not detected by αL78 antibody on the western blot (Fig. 5A, Control, WB: αL78), suggesting that heat treatment can trigger the proteolytic modification at the L78 insertion of Pho1 in sweet potato roots. Interestingly, a different form of the 20S proteasome with higher mobility in the control sample was observed after 12 h of incubation at 45°C (Fig. 5A, Control, WB: α20S).

To further verify whether proteasome is involved in the degradation of Pho1 at 45°C, the specific proteasome inhibitor MG132 was introduced to the experiments. The results showed that Pho1 degradation was efficiently inhibited by pretreating sweet potato roots with MG132, as the protein level of the intact Pho1 was almost no change within 2 h of incubation at 45°C (Fig. 5A, +MG132, Activity staining, WB: H7c, WB: αL78). Furthermore, the intact Pho1 remained clearly detectable even after 6–12 h of heat treatment by pretreating sweet potato roots with MG132 (Fig. 5A, +MG132, WB: H7c, WB: αL78), while they were largely degraded or not found in the control experiments (Fig. 5A, Control, WB: H7c, WB: αL78), indicating that the proteasome is involved in Pho1 degradation under heat treatment at 45°C. The data of the densitometry measurement of the intact Pho1 level on the western blots detected by αL78 antibody in Figure 5A also clearly demonstrated that 70% of the L78 insertion was degraded in the control sample after 2 h of incubation at 45°C. In contrast, only <5% of the L78 insertion was degraded in the MG132-pretreated sample after 2 h of incubation at 45°C (Fig. 5B), indicating that the proteasome plays a major role in the degradation of L78 insertion during heat treatment.

The proteolytic activity of the 20S proteasome toward the synthetic substrate Suc-LLVY-AMC was also measured in the experiments. The 20S proteasome activity in the control samples increased greater than two fold after 2 h of incubation at 45°C, and further increased greater than six fold after 12 h of incubation (Fig. 5C, Control). The results also showed that 50–70% of the 20S proteasome activity in the MG132-pretreated samples was inhibited within 2 h of incubation at 45°C (Fig. 5C, +MG132). However, the 20S proteasome activity was no longer efficiently inhibited by MG132 after 6 h of heat treatment, indicating that MG132 may become invalid under the high temperature and longer incubation time (Fig. 5C). Therefore, the Pho1 degradation began to be observed after 6 h of incubation at 45°C even though the sweet potato roots have been pretreated with MG132 (Fig. 5A, +MG132).

Although some studies have reported that sweet potato roots can grow well at 35°C as well as 40°C and had heat tolerance ranging between 35–48°C in summer [38], [39], we still consider the present experiments *in vitro* biochemical approaches because sweet potato roots have been cut into small discs, instead of growing under the soil, for performing the heat treatment experiments.

### The Proteolytically Modified Pho1 Showed Lower Affinity toward Glc-1-P

To determine whether the catalytic properties of Pho1 might change due to the proteolytic modification, we purified the intact Pho1 from sweet potato roots and the degraded Pho1 (Pho1_d_) from heat-treated sweet potato roots (Fig. S1). Enzyme kinetic parameters were determined by incubating Pho1 or Pho1_d_ with soluble starch and Glc-1-P to observe the kinetics of starch synthesis (Fig. S2). While Pho1 and Pho1_d_ exhibited similar kinetic behavior, Pho1 displayed higher affinity toward Glc-1-P (*K*_m_  =  0.7 mM) than Pho1_d_ (*K*_m_  =  1.4 mM) (Table 1). Consequently, Pho1 has higher catalytic activity (*K*_cat_/*K*_m_  =  20.86 mM^−1^ s^−1^) than Pho1_d_ (9.83 mM^−1 ^s^−1^) in the direction of starch synthesis (Table 1).

**Table 1:** Enzyme kinetic analysis of intact Pho1 and degraded Pho1 (Pho1_d_) in the starch synthesizing direction.

	*K*_m_			*K*_cat_ (sec^−1^)			*K*_cat_/*K*_m_	
	Soluble starch(%, w/v)	Glc-1-P(mM)		Fixed[Glc-1-P]^a^	Fixed[soluble starch]^b^		Fixed[Glc-1-P]^a^	Fixed[soluble starch]^b^
Pho1	0.11 ± 0.02	0.73 ± 0.02		13.04 ± 1.47	15.23 ± 0.15		118.55	20.86
Pho1_d_	0.12 ± 0.01	1.36 ± 0.06		12.79 ± 0.36	13.37 ± 0.33		106.58	9.83

Pho1_d_ was purified from sweet potato root discs that were incubated at 45°C for 36 h. Values are mean ± S.D. from three independent experiments.

a [Glc-1-P]  =  9.4 mM.

b [Soluble starch]  =  0.35%.

## Discussion

A canonical process for proteasome-mediated proteolytic modification of cellular proteins includes ubiquitination and the 19S proteasome activator [40]. However, evidence has established that some cellular proteins can be degraded without ubiquitination [37], [41], [42], and certain proteins appear to require only the 20S proteasome for turnover [36], [43]. Several studies also suggest that 20S proteasomes cleave substrates at disordered regions and outside structured domains [36], [42], [44]–[48]. In the present study, our data revealed that 20S proteasome is involved in regulating the stability and function of Pho1.

Pho1 undergoes proteolytic modification in mature potato tubers [13], sweet potato roots [24], and the developing mungbean [49]. However, this post-translational modification of Pho1 has not been observed in rice or maize endosperm [10], [50]. We speculate that the L78 from various plant species might have different sequence features. Multiple alignment of the L78 amino acid sequences from sweet potato and other plants showed that L78 from sweet potato shares 47%, 34% and 32% sequence identity with potato, rice and maize, respectively (Fig. S3). Interestingly, the degradation of Pho1 in potato tubers also occurred upon heat treatment (Fig. S4), implying that the discrepancy among different plant species may be due to the differences in the L78 sequences. However, it cannot be ruled out that the proteolytic modification of Pho1 is tissue-specific since plants have many specialized tissues which perform very different functions.

In the present study, an unusual high temperature at 45°C and a long incubation time for up to 12 hours were applied to examine the degradation of Pho1 proteins (Fig. 5). In the experiments, MG132 cannot completely inhibit Pho1 degradation. Thus, we cannot exclude the possibility that other proteases may also involve in heat-induced proteolysis of Pho1. Furthermore, MG132 is a peptide aldehyde which would not be stable for up to 12 hours at 45°C. Thus, a well-inhibited degradation reaction may be no longer inhibited when MG132 became invalid under the high temperature and longer incubation time. As a concern of that, the heat treatment procedure should be carefully stated as an *in vitro* experiment.

Although we found that Pho1 can be degraded by the 20S proteasome under a stringent heat stress condition, the signaling mechanism which initiates the degradation progress remains unclear. Phosphorylation was implicated as part of a regulation in targeting protein for ubiquitination and proteasomal degradation. I kappa B alpha is phosphorylated in response to various signals at two specific N-terminal Ser-32 and Ser-36; this leads to its ubiquitination and subsequent degradation [51]–[52]. Phosphorylation of cytosolic pyruvate kinase which undergoes a novel C-terminal proteolytic processing in developing soybean seeds is linked to its degradation via the ubiquitin-proteasome pathway [53]. The Akt Ser-473 phosphorylation promotes a Lys-48-linked polyubiquitination of Akt, resulting in its rapid proteasomal degradation [54]. Phosphorylation of sucrose synthase in maize leaves is part of a mechanism that targets sucrose synthase for proteasome-dependent degradation [34], [35]. On the contrary, a recent study showed that phosphorylation of alpha-synuclein is targeted to the proteasome pathway in a ubiquitin-independent manner [55]. Our previous study showed that phosphorylation of Pho1 at Ser-527 on the L78 insertion loop is more susceptible to proteolytic modification than normal Pho1 *in vitro* [26]. However, the 19S regulatory particles and ubiquitin were not found in the mass spectroscopic results of the HX complex (Fig. 2). Therefore, the possibility that phosphorylation serves as the signal for Pho1 degradation by ubiquitination and 26S proteasome is still elusive.

Starch branching enzymes have been observed to interact with Pho1 in a phosphorylation-dependent manner in the amyloplast, indicating that Pho1 is involved in starch synthesis [25]. Here we found that the 20S proteasome binds to the N-terminal domain and the L78 insertion of Pho1 (Fig. 4). The proteolytically modified Pho1 displays lower affinity toward Glc-1-P (Table 1; Fig. S2), suggesting that the catalytic direction of the degraded Pho1 is shifted in favor of starch phosphorolysis. As Pho1 catalyzes the reversible phosphorolysis of starch, we speculated that binding of Pho1 to the proteasome machinery or starch branching enzymes may mediate its biological role in modifying the structure of amylopectin via phosphorolysis or in operating the glucan formation, respectively. Therefore, this study raised an important question regarding whether the 20S proteasome and starch branching enzymes may bind to the same region of Pho1. If binding of starch branching enzymes to Pho1 inhibits the 20S proteasome binding, or vice versa, it may indicate that regulation of the catalytic direction of Pho1 can be achieved by association with different binding partners.

A handful of proteins regulated by the proteasome are cleaved internally and only partially processed. The NF-kappaB1 precursor p105 was cleaved into a functional p50 product by the 20S proteasome [56]. Glyceraldehyde-3-phosphate dehydrogenase was proteolytically modified by the 20S proteasome upon exposure to low doses of peroxynitrite [57]. Here we showed that the 20S proteasome-mediated degradation removes the L78 insertion loop of Pho1 and generates Pho1_d_ with lower affinity toward Glc-1-P (Table 1). Important questions remain regarding why some plants need two distinct functional properties of Pho1 in amyloplasts. Recent studies proposed that intact Pho1 has an essential role in the glucan initiation process by using Glc-1-P as a substrate to synthesize glucan primers [3], [48]. Our unpublished data (Chen et al.) also suggested that Pho1 with intact P110 can use Glc-1-P as the only substrates to produce glucan primers. However, based on the high orthophosphate/Glc-1-P ratio in amyloplasts, other research proposed that Pho1 might contribute to starch synthesis by modifying the structure of amylopectin by phosphorolysis [25]. Therefore, proteolytic modification may be an efficient way to regulate the catalytic direction of Pho1 in certain plants. We speculate that Pho1 with intact P110 may primarily play a role in the glucan initiation process by catalyzing reactions in the synthesis direction, while Pho1_d_ with proteolytic fragments (F50s) might mainly play a role in the phosphorolysis direction.

It is well established that the 26S proteasome is essential for the degradation of misfolded proteins and the elevated levels of the free 20S proteasome lead to increased tolerance to oxidative stress [58]–[60]. In the present study, we found that the activity of the 20S proteasome in sweet potato roots was induced by heat treatment at 45°C (Fig. 5C). The heat treatment also promoted the degradation of Pho1 by the proteasome (Fig. 5A). The proteolytically modified Pho1 (Pho1_d_) purified from heat-treated sweet potato roots displayed lower affinity toward Glc-1-P (Table 1), suggesting that Pho1_d_ prefers to catalyze the enzyme reaction in the direction of starch phosphorolysis. In this case, the levels of Glc-1-P were increased. Therefore, it seems likely that Pho1 degradation and the subsequent impact on its catalytic direction are also involved in heat stress response. It has been suggested that hexose phosphates supplied by phosphorylase can be the substrates for the oxidative pentose phosphate (OPP) pathway inside the chloroplast at night [22]. We propose that once Glc-1-P is converted to Glc-6-P, the OPP pathway can utilize Glc-6-P to provide reducing power (NADPH) for many biological reactions and control the levels of reactive oxygen intermediates.

Oxidatively modified proteins can form large aggregates because of increased surface hydrophobicity. Most oxidatively damaged proteins appear to undergo selective proteolysis, primarily by the proteasome. Contrary to earlier popular belief that most proteasomal degradation is conducted by the 26S proteasome with substrate ubiquitination, it has been shown that oxidized proteins are degraded without ubiquitin conjugation (or ATP hydrolysis) possibly by the 20S proteasome, and this degradation is still inhibited by proteasome inhibitors [61]. As noted above, the degradation of the L78 insertion loop of Pho1 in sweet potato roots was markedly promoted under heat treatment at 45°C (Fig. 5). Thus, the function of the insertion loop may serve as a "temperature switch" for regulating the catalytic direction of Pho1. Notably, this process is regulated through the partial truncation of the L78 insertion loop by the proteasome. Therefore, it is possible that Pho1 and the 20S proteasome are directly or indirectly involved in the cellular response for counteracting the effects induced by certain environmental stresses, such as heat stress, to minimize potential oxidative damage.

It was proposed that the hydrophobic patches of oxidized proteins bind to the α-subunits of the 20S proteasome, thereby opening the substrate channel [62], [63]. The L78 insertion loop has been predicted as a disordered region outside the main structure of Pho1 [24], [64]. Our data showed that the N-terminal domain and the L78 insertion loop of Pho1 interact with the 20S proteasome (Fig. 4). The highly flexible loop region may not require complete gate opening controlled by the 19S regulatory particles for entry into the catalytic core. Although the physical properties of the L78 insertion loop remains unclear, our data suggest that L78 plays certain roles in supporting this unique mode of Pho1 processing and catalytic regulation.

## Materials and Methods

### Plant Materials and Chemicals

Mature sweet potato roots [*Ipomoea batatas* (L.) Lam. cv Tainong 57] were collected from a local farm near Taipei, Taiwan. Chemicals were purchased from Sigma-Aldrich Co. unless otherwise noted.

### Assays of the Protein Content and Pho1 Activity

Protein content was determined by the dye-binding method [65] using the microassay system from Bio-Rad (Protein Assay Kit). Bovine serum albumin was used as the standard. The assays for Pho1 activity in the synthetic directions were carried out according to the procedures described previously [11], [24].

### Gel Electrophoresis and Immunoblotting

SDS-PAGE and native PAGE were performed according to the method described by Laemmli [66] with a slight modification. After electrophoresis, proteins were either stained with Coomassie Brilliant Blue R-250 or transferred to the PVDF membrane (Immobilon-P, Millipore) for western blotting. The protocols are described in detail in the previous report [67].

### In-gel Pho1 Activity Assay

Samples were first separated on a 6% native PAGE. After electrophoresis, the gel was incubated with the reaction buffer containing 20 mM MES-KOH (pH 5.9), 1.2% soluble starch (Nacalai Tesque, 321–22, Kyoto, Japan) and 32 mM Glc-1-P [68] at 37°C for 2 h. The zymogram was detected using iodine staining.

### Size-exclusion Chromatography

Sweet potato roots (150 g) were homogenized with buffer A (50 mM Tris-HCl, pH 7.4) containing 1% polyvinylpolypyrrolidone in a Waring blender followed by centrifugation at 48,000 x*g* for 30 min. The supernatant was separated on a Sephacryl S-300 column at 4°C. The fractions were separated by 6% native PAGE and analyzed by Coomassie staining, Pho1 activity staining, or western blotting with α20S antibody. The chromatography column was calibrated by using gel filtration standard (Bio-Rad), containing thyroglobulin (bovine) 670 kDa, γ-globulin (bovine) 158 kDa, ovalbumin (chicken) 44 kDa, myoglobin (horse) 17 kDa, and vitamin B_12_ 1.35 kDa.

### Purification of High Molecular Weight Pho1 Complex (HX)

Sweet potato roots (500 g) were homogenized with buffer A (50 mM Tris-HCl, pH 7.4) containing 1% polyvinylpolypyrrolidone in a Waring blender. The crude extract was separated on a Sephacryl S-300 column as described previously. The fractions containing Pho1 activities were determined by in-gel Pho1 activity assay. The high molecular weight fractions with Pho1 activity (HX) were collected and loaded on a DEAE-Sephacel column following the elution step with a linear gradient of NaCl (0.15∼0.5 M) in buffer A. The fractions containing HX were collected and concentrated by ultrafiltration (Microcon YM30, Millipore, Bedford, MA, USA). All purification procedures were carried out at 4°C.

### Identification of Proteins by Q-TOF-MS

Selected protein targets were excised from the Coomassie-stained gel according to the standard MS sample preparation protocol [69]. In-gel digestion of proteins was carried out using MS-grade Trypsin Gold (Promega, Madison, WI, USA) at 37°C overnight. Tryptic cleavage products were extracted with 10 µl of Milli-Q water and 100 µl of 50% acetonitrile/5% TFA, and then dried in a vacuum concentrator at room temperature. The samples were dissolved in 10 µl of 50% acetonitrile/0.1% formic acid solution and then subjected to analysis on a C18 column (75 µm i.d., 10 cm in length, MST, Taiwan) with a linear gradient of 5∼50% acetonitrile in 0.1% formic acid in the CapLC system (Waters, Milford, MA). The separated peptides were online analyzed under positive survey scan mode by a nano-ESI Q-TOF (Micromass, UK) instrument. The MS data were analyzed by using the MASCOT searching engine (www.matrixscience.com).

### Purification of Pho1 and the 20S Proteasome

Pho1 was purified according to a method described previously [24]. The 20S proteasome was purified according to the method described by Chang et al. [20] with a slight modification. Sweet potato roots (400 g) were homogenized with buffer A (50 mM Tris-HCl, pH 7.4) containing 1% polyvinylpolypyrrolidone in a Waring blender. The crude extract was further fractionated by ammonium sulfate precipitation. The protein precipitate at ammonium sulfate saturation between 45% and 80% was collected and dissolved in a minimal amount of buffer A. The sample was dialyzed against buffer B (buffer A containing 0.15 M NaCl) overnight and then applied on a DEAE-Sephacel column following the elution step with a linear gradient of NaCl (0.15–0.5 M) in buffer A. The fractions containing the 20S proteasome were further purified by a Sephacryl S-300 column and a subsequent phenyl-Sepharose column following the elution step with a linear gradient of ammonium sulfate (0.5∼0 M) in buffer A. For a better separation result, a second size-exclusion chromatography by Superose 6 HR 10/300 (Pharmacia) was applied to the purification procedure and the fractions containing the 20S proteasome were subjected to a preparative gel electrophoresis (6% native PAGE). The highly purified 20S proteasome was obtained by the electroelution of the gel blocks with the Little Blue Tank (ISCO Inc., Lincoln, Nebraska, USA). All purification procedures were carried out at 4°C.

### Preparation of Antibodies

The conventional rabbit antiserum against the 20S proteasome (α20S) was generated by immunizing rabbits with the purified 20S proteasome from sweet potato roots. The mouse conventional antiserum against Pho1 was produced from mice immunized with purified Pho1 from sweet potato roots. Mouse monospecific antibody anti-L1 against the peptide sequence TKPKETSIVDPSEEVEVSG on the L78 insertion, and mouse monoclonal antibodies H7c and J3b, were prepared as described by Chen et al. [24]. The mouse monoclonal antibody anti-L78 was produced as described previously [26].

### Immunoprecipitation

Antibody crosslinking procedures were performed according to the method described by Sisson and Castor [70] with a slight modification. Monoclonal antibody H7c and anti-20S proteasome antibody (α20S) were crosslinked to Protein A Sepharose CL-4B beads (Amersham Biosciences) with 50 mM dimethyl pimelimidate·2 HCl (DMP, Pierce) and 50 mM dimethyl suberimidate·2 HCl (DMS, Pierce) in 0.2 M triethanolamide and 0.1 M sodium borate (pH 8.2) at room temperature for 30 min, respectively. Crosslinking reaction was terminated by incubating beads with 50 mM ethanolamine (pH 8.2) for 10 min. Beads were subsequently washed with 1 M glycine (pH 3) for 20 min to remove uncrosslinked antibodies and equilibrated in phosphate buffer saline (PBS). The whole cell extracts from sweet potato roots were incubated with the H7c-crosslinked and α20S-crosslinked Protein A Sepharose CL-4B beads at 4°C for 12 h, respectively. Beads were washed several times with PBS containing 0.05% Tween-20 (PBST). Bound proteins were eluted by SDS-PAGE sample buffer (1X) without containing a reducing agent and subsequently separated by 12.5% SDS-PAGE under the reducing condition.

### Two-dimensional Native/SDS Gel Electrophoresis

The first-dimension 6% native PAGE (0.75 mm thick) was run on a conventional slab gel system. The lanes on the gel slab were cut into individual strips, and then transferred to PVDF membranes for western blotting with specific antibodies. Alternatively, the strips were incubated with equilibration buffer (50 mM Tris-HCl, pH 8.0, 1% DTT, 2% SDS, 30% glycerol, 6 M urea, and 0.01% bromophenol blue) at 37°C for 30 min. Subsequently, the gel strips were placed onto the top of the stacking gel (4%) of the second-dimension 12.5% SDS-PAGE (1 mm thick). The electrophoresis was run at 150 V for 2 h. Proteins on the slab were transferred to PVDF membranes and immunostained with specific antibodies.

### Heat Treatment of Sweet Potato Root Discs

Sweet potato root discs (5 cm in diameter and approximately 0.5 mm thick) were cut from mature sweet potato roots in the laminar flow hood. The discs were first incubated with or without 100 µM MG132 (Sigma-Aldrich Co.) in 10 mM sterile MES-KOH (pH 6.5) buffer containing 60 mM sucrose at room temperature for 2 h. After the pretreatment, the discs were sealed in sterile plastic bags and incubated at 45°C in an oven for the indicated time intervals. At each time point, the discs were transferred quickly from the oven to the liquid nitrogen and then ground into powder in a mortar. The ground powder (0.4 g) was extracted with 200 µl of extraction buffer (100 mM Tris-HCl (pH 7.4), 5 mM EDTA, 10 mM β-mercaptoethanol, 20 mM NaF, 1 mM Na_3_VO_4_, 1% polyvinylpolypyrrolidone and 1 mM phenylmethylsulfonyl fluoride).

### Proteasome Activity Assay

Chymotrypsin-like activity of the 20S proteasome was determined by measuring the hydrolysis of the fluorogenic substrate N-succinyl-Leu-Leu-Val-Tyr-7-amino-4-methylcoumarin (Suc-LLVY-AMC, Sigma-Aldrich, Inc.) in 100 mM Tris (pH 7.6), 1 mM DTT, 0.1 mM Suc-LLVY-AMC and 0.02% SDS at 37°C for 30 min [35]. The fluorescence of the released amido-methyl coumarin (AMC) was measured with a DTX 880 Multimode Detector (Beckman Coulter, Fullerton, CA) at the excitation of 380 nm and the emission of 480 nm.

## Supporting Information

Figure S1 **Characterization of intact Pho1 (Pho1) and proteolytic modified Pho1 (Pho1d) by SDS-PAGE and western blot analysis.** Intact Pho1 (Pho1) was purified from sweet potato root discs which did not undergo heat treatment. Proteolytic modified Pho1 (Pho1d) was purified from sweet potato root discs which were incubated at 45°C for 36 h. Purified Pho1 or Pho1d (10 µg) was separated by 12.5% SDS-PAGE and analyzed by Coomassie Brilliant Blue R-250 (Coomassie) staining or western blot with H7c, J3b or αL78 antibody, respectively. P110 is the intact form of Pho1 revealing the molecular weight at 110 kDa. F50s are a group of proteolytic modified Pho1 revealing the molecular weight around 50 kDa. P110 was not observed in the Pho1d sample. Since αL78 did not display any cross-reaction with Pho1d, it reveals that Pho1d does not contain the L78 insertion.
(DOC)

Figure S2 **Determination of the enzyme kinetic parameters of intact Pho1 (Pho1) and proteolytic modified Pho1 (Pho1d).** (**A**) The double reciprocal plot using Glc-1-P (9.4 mM) as the limiting substrate. (**B**) The double reciprocal plot using soluble starch (0.35%) as the limiting substrate. Pho1, open circle (○); Pho1d, solid triangle (▴). Values are mean ± S.D. from three independent experiments.
(DOC)

Figure S3 **Multiple alignment of the L78 amino acid sequences from different plant species.** Multiple alignment of the L78 amino acid sequences of Pho1 from *Ipomoea batatas* (GenBank accession number, P27598.1), *Solanum tuberosum* (GenBank accession number, P04045.2), *Oryza sativa* (GenBank accession number, AAK15695.1) and *Zea mays* (GenBank accession number, CAB69360.1) was done by using ClustalW program. Identical residues conserved in all sequences were marked with asterisks (*). The conserved substitutions among different sequences were denoted as colons (:).
(DOC)

Figure S4 **Pho1 in potato tubers undergoes proteolytic degradation upon heat treatment.** Potato tubers discs (2 mm thickness) were incubated at 45°C for the indicated time periods up to 48 h. (**A**) The crude protein extracts of the discs (10 µg) were separated by 7.5% native PAGE and analyzed by Coomassie staining, Pho1 activity staining or western blot analysis with αPho1 polyclonal antibody, respectively. (**B**) Alternatively, the same samples above were subjected to 12.5% SDS-PAGE and analyzed by Coomassie or western blot analysis with αPho1, respectively.
(DOC)

## References

[1] Ball S, Guan HP, James M, Myers A, Keeling P (1996). From glycogen to amylopectin: a model for the biogenesis of the plant starch granule.. Cell.

[2] Dauvillee D, Chochois V, Steup M, Haebel S, Eckermann N (2006). Plastidial phosphorylase is required for normal starch synthesis in *Chlamydomonas reinhardtii*.. Plant J.

[3] Satoh H, Shibahara K, Tokunaga T, Nishi A, Tasaki M (2008). Mutation of the plastidial alpha-glucan phosphorylase gene in rice affects the synthesis and structure of starch in the endosperm.. Plant Cell.

[4] Schupp N, Ziegler P (2004). The relation of starch phosphorylases to starch metabolism in wheat.. Plant Cell Physiol.

[5] Sonnewald U, Basner A, Greve B, Steup M (1995). A second L-type isozyme of potato glucan phosphorylase: cloning, antisense inhibition and expression analysis.. Plant Mol Biol.

[6] Hanes CS (1940). The breakdown and synthesis of starch by an enzyme system from pea seeds.. Proc R Soc Lond B.

[7] Hanes CS (1940). The reversible formation of starch from glucose-1-phosphate catalysed by potato phosphorylase.. Proc R Soc Lond B.

[8] Shimomura S, Nagai M, Fukui T (1982). Comparative glucan specificities of two types of spinach leaf phosphorylase.. J Biochem.

[9] Steup M, Preiss J (1988). Starch degradation.. The Biochemistry of Plants Vol 14 Carbohydrates.

[10] Yu Y, Mu HH, Wasserman BP, Carman GM (2001). Identification of the maize amyloplast stromal 112-kD protein as a plastidic starch phosphorylase.. Plant Physiol.

[11] Mori H, Tanizawa K, Fukui T (1993). A chimeric alpha-glucan phosphorylase of plant type L and H isozymes. Functional role of 78-residue insertion in type L isozyme.. J Biol Chem.

[12] Albrecht T, Koch A, Lode A, Greve B, Schneider-Mergener J (2001). Plastidic (Pho1-type) phosphorylase isoforms in potato (*Solanum tuberosum* L.) plants: expression analysis and immunochemical characterization.. Planta.

[13] Brisson N, Giroux H, Zollinger M, Camirand A, Simard C (1989). Maturation and subcellular compartmentation of potato starch phosphorylase.. Plant Cell.

[14] Duwenig E, Steup M, Kossmann J (1997). Induction of genes encoding plastidic phosphorylase from spinach (*Spinacia oleracea* L.) and potato (*Solanum tuberosum* L.) by exogenously supplied carbohydrates in excised leaf discs.. Planta.

[15] Mingo-Castel AM, Young RE, Smith OE (1976). Kinetin-induced tuberization of potato *in vitro*: on the mode of action of kinetin.. Plant Cell Physiol.

[16] St-pierre B, Brisson N (1995). Induction of the plastidic starch-phosphorylase gene in potato storage sink tissue.. Planta.

[17] Liu TT, Shannon JC (1981). Measurement of metabolites associated with nonaqueously isolated starch granules from immature *Zea mays* L. endosperm.. Plant Physiol.

[18] Baun LC, Palmiano EP, Perez CM, Juliano BO (1970). Enzymes of starch metabolism in the developing rice grain.. Plant Physiol.

[19] Ohdan T, Francisco PB, Sawada T, Hirose T, Terao T (2005). Expression profiling of genes involved in starch synthesis in sink and source organs of rice.. J Exp Bot.

[20] Chang SC, Lin PC, Chen HM, Wu JS, Juang RH (2000). The isolation and characterization of Chaperonin 60 from sweet potato roots - Involvement of the chaperonins in starch biosynthesis.. Bot Bull Academia Sin.

[21] Van Berkel J, Conrads-Strauch J, Steup M (1991). Glucan-phosphorylase forms in cotyledons of *Pisum sativum* L.: Localization, developmental change, *in-vitro* translation, and processing.. Planta.

[22] Zeeman SC, Thorneycroft D, Schupp N, Chapple A, Weck M (2004). Plastidial alpha-glucan phosphorylase is not required for starch degradation in Arabidopsis leaves but has a role in the tolerance of abiotic stress.. Plant Physiol.

[23] Chiang CL, Lu YL, Juang RH, Lee PD, Su JC (1991). Native and degraded forms of sweet potato starch phosphorylase.. Agric Biol Chem.

[24] Chen HM, Chang SC, Wu CC, Cuo TS, Wu JS (2002). Regulation of the catalytic behaviour of L-form starch phosphorylase from sweet potato roots by proteolysis.. Physiol Plant.

[25] Tetlow IJ, Wait R, Lu Z, Akkasaeng R, Bowsher CG (2004). Protein phosphorylation in amyloplasts regulates starch branching enzyme activity and protein-protein interactions.. Plant Cell.

[26] Young GH, Chen HM, Lin CT, Tseng KC, Wu JS (2006). Site-specific phosphorylation of L-form starch phosphorylase by the protein kinase activity from sweet potato roots.. Planta.

[27] Coux O, Tanaka K, Goldberg AL (1996). Structure and functions of the 20S and 26S proteasomes.. Annu Rev Biochem.

[28] Santt O, Pfirrmann T, Braun B, Juretschke J, Kimmig P (2008). The yeast GID complex, a novel ubiquitin ligase (E3) involved in the regulation of carbohydrate metabolism.. Mol Biol Cell.

[29] Vierstra RD (2009). The ubiquitin-26S proteasome system at the nexus of plant biology.. Nat Rev Mol Cell Biol.

[30] Bulteau AL, Verbeke P, Petropoulos I, Chaffotte AF, Friguet B (2001). Proteasome inhibition in glyoxal-treated fibroblasts and resistance of glycated glucose-6-phosphate dehydrogenase to 20 S proteasome degradation *in vitro*.. J Biol Chem.

[31] Hung GC, Brown CR, Wolfe AB, Liu J, Chiang HL (2004). Degradation of the gluconeogenic enzymes fructose-1,6-bisphosphatase and malate dehydrogenase is mediated by distinct proteolytic pathways and signaling events.. J Biol Chem.

[32] Saitoh F, Araki T (2010). Proteasomal degradation of glutamine synthetase regulates schwann cell differentiation.. J Neurosci.

[33] Brown CR, Chiang HL (2009). A selective autophagy pathway that degrades gluconeogenic enzymes during catabolite inactivation.. Commun Integr Biol.

[34] Hardin SC, Huber SC (2004). Proteasome activity and the post-translational control of sucrose synthase stability in maize leaves.. Plant Physiol Biochem.

[35] Hardin SC, Tang GQ, Scholz A, Holtgraewe D, Winter H (2003). Phosphorylation of sucrose synthase at serine 170: occurrence and possible role as a signal for proteolysis.. Plant J.

[36] Baugh JM, Viktorova EG, Pilipenko EV (2009). Proteasomes can degrade a significant proportion of cellular proteins independent of ubiquitination.. J Mol Biol.

[37] Li X, Amazit L, Long W, Lonard DM, Monaco JJ (2007). Ubiquitin- and ATP-independent proteolytic turnover of p21 by the REGgamma-proteasome pathway.. Mol Cell.

[38] Sattelmacher B, Marschner H, Kuhne R (1990). Effects of the temperature of the rooting zone on the growth and development of roots of potato (*Solanum tuberosum* L.). Ann. Bot..

[39] Weng JH, Lai MF (2005). Estimating heat tolerance among plant species by two chlorophyll fluorescence parameters.. Photosynthetica.

[40] Varshavsky A (2005). Regulated protein degradation.. Trends Biochem Sci.

[41] Asher G, Tsvetkov P, Kahana C, Shaul Y (2005). A mechanism of ubiquitin-independent proteasomal degradation of the tumor suppressors p53 and p73.. Genes Dev.

[42] Orlowski M, Wilk S (2003). Ubiquitin-independent proteolytic functions of the proteasome.. Arch Biochem Biophys.

[43] Asher G, Reuven N, Shaul Y (2006). 20S proteasomes and protein degradation "by default".. Bioessays.

[44] Baugh JM, Pilipenko EV (2004). 20S proteasome differentially alters translation of different mRNAs via the cleavage of eIF4F and eIF3.. Mol Cell.

[45] Grune T, Merker K, Sandig G, Davies KJ (2003). Selective degradation of oxidatively modified protein substrates by the proteasome.. Biochem Biophys Res Commun.

[46] Hoyt MA, Coffino P (2004). Ubiquitin-free routes into the proteasome.. Cell Mol Life Sci.

[47] Liu CW, Corboy MJ, DeMartino GN, Thomas PJ (2003). Endoproteolytic activity of the proteasome.. Science.

[48] Sorokin AV, Selyutina AA, Skabkin MA, Guryanov SG, Nazimov IV (2005). Proteasome-mediated cleavage of the Y-box-binding protein 1 is linked to DNA-damage stress response.. EMBO J.

[49] Ko YT, Chang JY, Lee YT, Wu YH (2005). The identification of starch phosphorylase in the developing mungbean (*Vigna radiata* L.).. J Agric Food Chem.

[50] Hwang SK, Nishi A, Satoh H, Okita TW (2010). Rice endosperm-specific plastidial alpha-glucan phosphorylase is important for synthesis of short-chain malto-oligosaccharides.. Arch Biochem Biophys.

[51] Chen Z, Hagler J, Palombella VJ, Melandri F, Scherer D (1995). Signal-induced site-specific phosphorylation targets I kappa B alpha to the ubiquitin-proteasome pathway. Genes Dev.. 9,.

[52] Alkalay I, Yaron A, Hatzubai A, Orian A, Ciechanover A (1995). Stimulation-dependent I kappa B alpha phosphorylation marks the NF-kappa B inhibitor for degradation via the ubiquitin-proteasome pathway.. Proc Natl Acad Sci U S A. 92,.

[53] Tang GQ, Hardin SC, Dewey R, Huber SC (2003). A novel C-terminal proteolytic processing of cytosolic pyruvate kinase, its phosphorylation and degradation by the proteasome in developing soybean seeds.. Plant J.

[54] Wu YT, Ouyang W, Lazorchak AS, Liu D, Shen HM (2011). mTOR complex 2 targets Akt for proteasomal degradation via phosphorylation at the hydrophobic motif. J Biol Chem.. 286,.

[55] Machiya Y, Hara S, Arawaka S, Fukushima S, Sato H (2010). Phosphorylated alpha-synuclein at Ser-129 is targeted to the proteasome pathway in a ubiquitin-independent manner.. J Biol Chem.

[56] Moorthy AK, Savinova OV, Ho JQ, Wang VY, Vu D (2006). The 20S proteasome processes NF-kappaB1 p105 into p50 in a translation-independent manner.. EMBO J.

[57] Buchczyk DP, Grune T, Sies H, Klotz LO (2003). Modifications of glyceraldehyde-3-phosphate dehydrogenase induced by increasing concentrations of peroxynitrite: early recognition by 20S proteasome.. Biol Chem.

[58] Shringarpure R, Grune T, Mehlhase J, Davies KJ (2003). Ubiquitin conjugation is not required for the degradation of oxidized proteins by proteasome.. J Biol Chem.

[59] Davies KJ (2001). Degradation of oxidized proteins by the 20S proteasome.. Biochimie.

[60] Kurepa J, Toh EA, Smalle JA (2008). 26S proteasome regulatory particle mutants have increased oxidative stress tolerance.. Plant J.

[61] Poppek D, Grune T (2006). Proteasomal defense of oxidative protein modifications.. Antioxid Redox Signal.

[62] Pacifici RE, Kono Y, Davies KJ (1993). Hydrophobicity as the signal for selective degradation of hydroxyl radical-modified hemoglobin by the multicatalytic proteinase complex, proteasome. J Biol Chem.. 268,.

[63] Grune T, Merker K, Sandig G, Davies KJ (2003). Selective degradation of oxidatively modified protein substrates by the proteasome. Biochem Biophys Res Commun.. 305,.

[64] Nakano K, Fukui T (1986). The complete amino acid sequence of potato alpha-glucan phosphorylase. J Biol Chem.. 261,.

[65] Bradford MM (1976). A rapid and sensitive method for the quantitation of microgram quantities of protein utilizing the principle of protein-dye binding.. Anal Biochem.

[66] Laemmli UK (1970). Cleavage of structural proteins during the assembly of the head of bacteriophage T4.. Nature.

[67] Wang HC, Wu JS, Chia JC, Yang CC, Wu YJ (2009). Phytochelatin synthase is regulated by protein phosphorylation at a threonine residue near its catalytic site.. J Agric Food Chem.

[68] Chang TC, Lee SC, Su JC (1987). Sweet potato starch phosphorylase-purification and characterization.. Agric Biol Chem.

[69] Walker JM (2002). The Protein Protocols Handbook..

[70] Sisson TH, Castor CW (1990). An improved method for immobilizing IgG antibodies on protein A-agarose.. J Immunol Methods.

