# Supplementary material for: Plastidial Starch Phosphorylase in Sweet Potato Roots Is Proteolytically Modified by Protein-Protein Interaction with the 20S Proteasome
Source: PLoS One. 2012 Apr 10;7(4):e35336. doi: 10.1371/journal.pone.0035336 (PMC3323651; doi:10.1371/journal.pone.0035336)
Supplement: Figure S1 — Characterization of intact Pho1 (Pho1) and proteolytic modified Pho1 (Pho1d) by SDS-PAGE and western blot analysis. Intact Pho1 (Pho1) was purified from sweet potato root discs which did not undergo heat treatment. Proteolytic modified Pho1 (Pho1d) was purified from sweet potato root discs which were incubated at 45°C for 36 h. Purified Pho1 or Pho1d (10 µg) was separated by 12.5% SDS-PAGE and analyzed by Coomassie Brilliant Blue R-250 (Coomassie) staining or western blot with H7c, J3b or αL78 antibody, respectively. P110 is the intact form of Pho1 revealing the molecular weight at 110 kDa. F50s are a group of proteolytic modified Pho1 revealing the molecular weight around 50 kDa. P110 was not observed in the Pho1d sample. Since αL78 did not display any cross-reaction with Pho1d, it reveals that Pho1d does not contain the L78 insertion. (DOC) [file pone.0035336.s001.doc]

**Figure S1. Characterization of intact Pho1 (Pho1) and proteolytic modified Pho1 (Pho1_d_) by SDS-PAGE and western blot analysis.**

Intact Pho1 (Pho1) was purified from sweet potato root discs which did not undergo heat treatment. Proteolytic modified Pho1 (Pho1_d_) was purified from sweet potato root discs which were incubated at 45ºC for 36 h. Purified Pho1 or Pho1_d_ (10 μg) was separated by 12.5% SDS-PAGE and analyzed by Coomassie Brilliant Blue R-250 (Coomassie) staining or western blot with H7c, J3b or αL78 antibody, respectively. P110 is the intact form of Pho1 revealing the molecular weight at 110 kDa. F50s are a group of proteolytic modified Pho1 revealing the molecular weight around 50 kDa. P110 was not observed in the Pho1_d_ sample. Since αL78 did not display any cross-reaction with Pho1_d_, it reveals that Pho1_d_ does not contain the L78 insertion.
