# Supplementary material for: Plastidial Starch Phosphorylase in Sweet Potato Roots Is Proteolytically Modified by Protein-Protein Interaction with the 20S Proteasome
Source: PLoS One. 2012 Apr 10;7(4):e35336. doi: 10.1371/journal.pone.0035336 (PMC3323651; doi:10.1371/journal.pone.0035336)
Supplement: Figure S2 — Determination of the enzyme kinetic parameters of intact Pho1 (Pho1) and proteolytic modified Pho1 (Pho1d). (A) The double reciprocal plot using Glc-1-P (9.4 mM) as the limiting substrate. (B) The double reciprocal plot using soluble starch (0.35%) as the limiting substrate. Pho1, open circle (○); Pho1d, solid triangle (▴). Values are mean ± S.D. from three independent experiments. (DOC) [file pone.0035336.s002.doc]

**Figure S2. Determination of the enzyme kinetic parameters of intact Pho1 (Pho1) and proteolytic modified Pho1 (Pho1_d_).**

(**A**) The double reciprocal plot using Glc-1-P (9.4 mM) as the limiting substrate. (**B**) The double reciprocal plot using soluble starch (0.35%) as the limiting substrate. Pho1, open circle (○); Pho1_d_, solid triangle (▲). Values are mean ± S.D. from three independent experiments.
