# Supplementary material for: Plastidial Starch Phosphorylase in Sweet Potato Roots Is Proteolytically Modified by Protein-Protein Interaction with the 20S Proteasome
Source: PLoS One. 2012 Apr 10;7(4):e35336. doi: 10.1371/journal.pone.0035336 (PMC3323651; doi:10.1371/journal.pone.0035336)
Supplement: Figure S3 — Multiple alignment of the L78 amino acid sequences from different plant species. Multiple alignment of the L78 amino acid sequences of Pho1 from Ipomoea batatas (GenBank accession number, P27598.1), Solanum tuberosum (GenBank accession number, P04045.2), Oryza sativa (GenBank accession number, AAK15695.1) and Zea mays (GenBank accession number, CAB69360.1) was done by using ClustalW program. Identical residues conserved in all sequences were marked with asterisks (*). The conserved substitutions among different sequences were denoted as colons (:). (DOC) [file pone.0035336.s003.doc]

**Figure S3. Multiple alignment of the L78 amino acid sequences from different plant species**

Multiple alignment of the L78 amino acid sequences of Pho1 from *Ipomoea batatas* (GenBank accession number, P27598.1), *Solanum tuberosum* (GenBank accession number, P04045.2), *Oryza sativa* (GenBank accession number, AAK15695.1) and *Zea mays* (GenBank accession number, CAB69360.1) was done by using ClustalW program. Identical residues conserved in all sequences were marked with asterisks (*). The conserved substitutions among different sequences were denoted as colons (**:**).
