# Supplementary material for: Plastidial Starch Phosphorylase in Sweet Potato Roots Is Proteolytically Modified by Protein-Protein Interaction with the 20S Proteasome
Source: PLoS One. 2012 Apr 10;7(4):e35336. doi: 10.1371/journal.pone.0035336 (PMC3323651; doi:10.1371/journal.pone.0035336)
Supplement: Figure S4 — Pho1 in potato tubers undergoes proteolytic degradation upon heat treatment. Potato tubers discs (2 mm thickness) were incubated at 45°C for the indicated time periods up to 48 h. (A) The crude protein extracts of the discs (10 µg) were separated by 7.5% native PAGE and analyzed by Coomassie staining, Pho1 activity staining or western blot analysis with αPho1 polyclonal antibody, respectively. (B) Alternatively, the same samples above were subjected to 12.5% SDS-PAGE and analyzed by Coomassie or western blot analysis with αPho1, respectively. (DOC) [file pone.0035336.s004.doc]

**Figure S4.** **Pho1 in potato tubers undergoes proteolytic degradation upon heat treatment**

Potato tubers discs (2 mm thickness) were incubated at 45ºC for the indicated time periods up to 48 h. (**A**) The crude protein extracts of the discs (10 μg) were separated by 7.5% native PAGE and analyzed by Coomassie staining, Pho1 activity staining or western blot analysis with αPho1 polyclonal antibody, respectively. (**B**) Alternatively, the same samples above were subjected to 12.5% SDS-PAGE and analyzed by Coomassie or western blot analysis with αPho1, respectively.
